# A Review of the Principles, Methodologies and Characteristics of Dry Eye Animal Models

**DOI:** 10.3390/ijms27156977

**Published:** 2026-08-03

**Authors:** Wenbo Dong, Liangyu Zhou, Yau Kei Chan, Hua Wang

**Affiliations:** 1 Eye Center of Xiangya Hospital, National Clinical Key Specialty of Ophthalmology, Hunan Key Laboratory of Ophthalmology, Central South University, Changsha 410083, China; 238112338@csu.edu.cn; 2 National Clinical Research Center for Geriatric Disorders, Xiangya Hospital, Central South University, Changsha 410028, China; 3 Department of Ophthalmology, Li Ka Shing Faculty of Medicine, The University of Hong Kong, Hong Kong SAR 999077, China; u3006804@connect.hku.hk

**Keywords:** dry eye, animal model, ocular surface disease, MGD, dry eye model

## Abstract

Dry eye disease is one of the most prevalent diseases on the ocular surface, which can cause discomfort and visual impairment in patients. As a chronic disorder, dry eye can be caused by various factors, including reduced tear film stability, increased tear osmotic pressure and inflammation response on the ocular surface. Due to its complex and still unclear pathogenesis, many challenges related to the diagnosis and treatment of dry eye must be addressed through animal studies. An ideal animal model for dry eye is essential to the research on the pathogenesis, diagnosis, and treatment of the disease. However, there is a lack of comprehensive systematic reviews evaluating diverse methodologies employed in the development of related animal models. This review summarizes the principles, methodologies, and characteristics of different dry eye animal models, aiming to provide references for selecting relevant dry eye models for drug modeling, lacrimal gland resection, meibomian gland dysfunction, autoimmune, and environmental modeling.

## 1. Introduction

**Figure 1:**
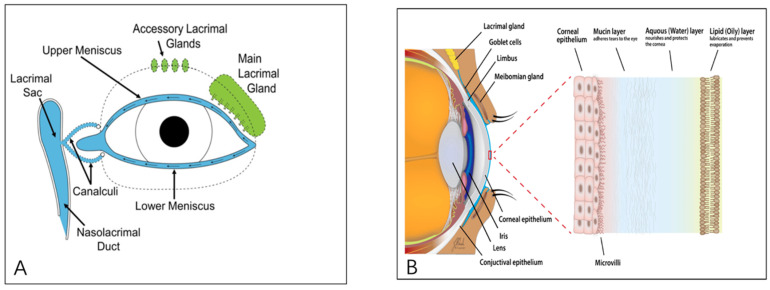
(**A**) Distribution of the main lacrimal gland, accessory lacrimal glands, and the lacrimal secretion system on the ocular surface. The dash line represents the projection of the anatomical edge of the eyelid. Reproduced from [6] with permission from Elsevier. Copyright 2010, Elsevier. (**B**) Schematic representation of the tear film layers: lipid layer, aqueous layer, and mucin layer. Reproduced from [7] with permission from the International Journal of Molecular Sciences. Copyright 2019, International Journal of Molecular Sciences.

Dry eye disease (DED) is one of the most common ocular surface disorders worldwide [1]. Recent large-scale epidemiological studies and meta-analyses indicate that its prevalence varies substantially depending on diagnostic criteria, age, and geographic region [2]. Symptom-based global prevalence estimates range from approximately 12% to 34% in adults, while sign-based estimates are generally lower [3]. It is a multifactorial chronic ocular surface disease characterized by instability of the tear film and disruption of the ocular surface microenvironment [4]. A central feature of its pathology is the loss of tear film homeostasis and subsequently abnormal tear quality, quantity, or dynamics [4]. The tear film consists of three layers: the superficial lipid layer secreted by meibomian glands, the middle aqueous layer produced by the lacrimal glands, and the inner mucin layer derived from conjunctival goblet cells (Figure 1). There are two sublayers in the middle aqueous layer, the superficial layer is a nonpolar lipid sublayer, while the inner layer is a polar lipid sublayer. The occurrence of dry eye is frequently associated with ocular surface inflammation, tissue damage, and neural abnormalities, leading to a variety of discomfort symptoms and/or visual impairment [5]. The investigation of dry-eye-related pathogenesis, as well as the assessment of diagnostic and therapeutic efficacy usually relies on animal models. Owing to the multifactorial nature of dry eye disease, the selection of an appropriate animal model that accurately reflects its development is crucial for elucidating disease mechanisms and evaluating novel therapeutic strategies. However, despite the establishment of numerous animal models for dry eye in recent years, comprehensive and up-to-date reviews critically assessing these approaches are still lacking. This article presents a systematic review of the methodologies employed to develop various animal models of dry eye, including pharmacological induction, lacrimal gland excision, induction of meibomian gland dysfunction (MGD), utilization of autoimmune mechanisms, and adaption of environmental factors. Furthermore, the advantages, limitations, and potential applications of these models are also discussed, offering insights into the criteria necessary for establishing an ideal animal model for dry eye research.

## 2. Drug-Induced Dry Eye Animal Models

**Figure 2:**
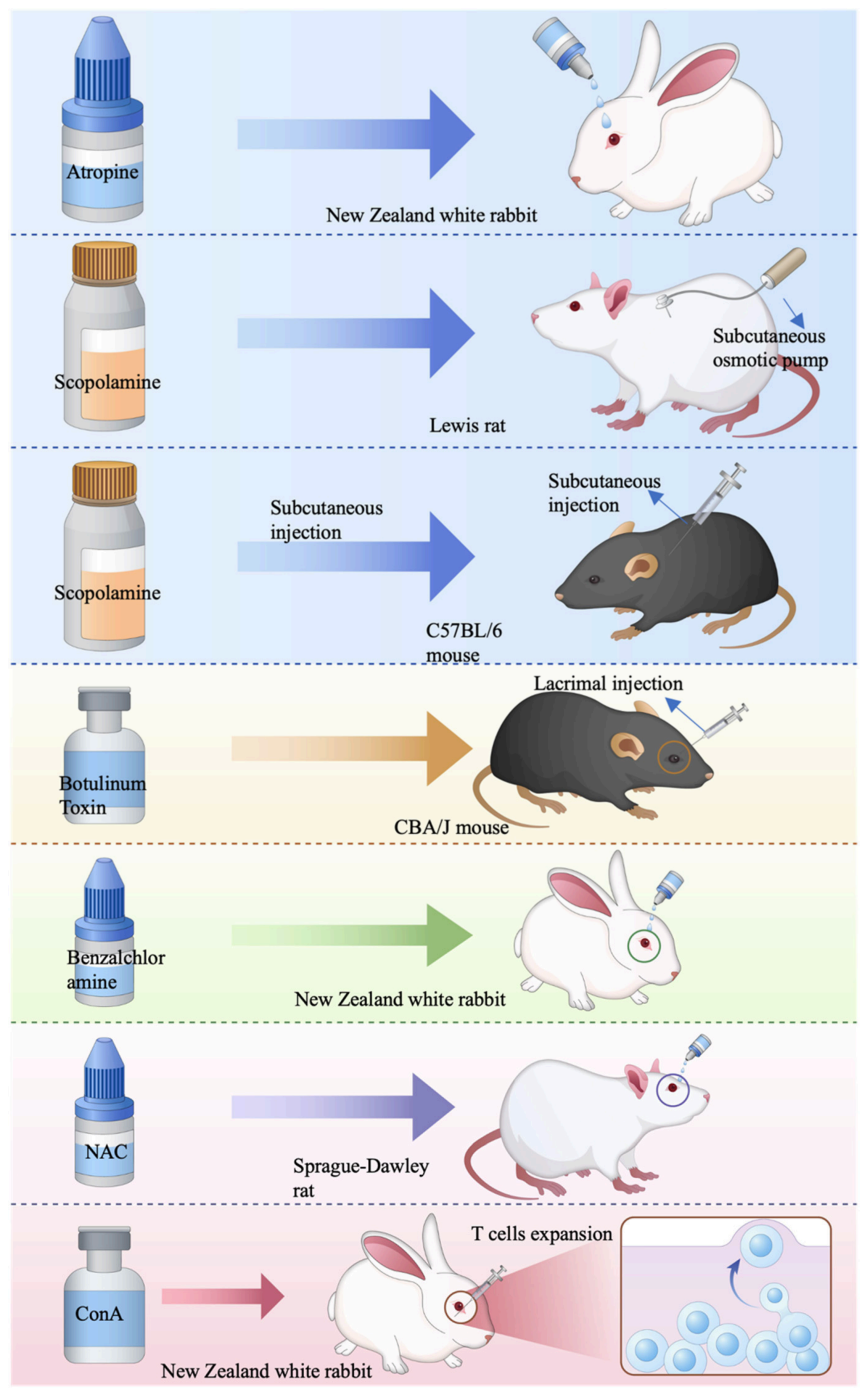
Illustration of drug-related dry eye animal models.

The molecular compounds that can induce dry eye on animals include acetylcholine receptor blockers [8,9,10], botulinum toxin (BTX) [11], benzalkonium chloride [12], N-acetylcysteine (NAC) [13] and Concanavalin A (ConA) [14]. However, most of these dry eye models are transient in nature, typically resulting in relatively short-duration manifestations of the disease. Additionally, these compounds may lead to the risk of toxicity when administered on the ocular surface. Despite the limitations, these molecule compounds are widely used due to their convenience and efficiency in constructing models. An overview of commonly employed drug-induced dry eye models is provided in Table 1 and Figure 2.

### 2.1. Models Based on Acetylcholine Receptor Blocking Agents

**Figure 3:**
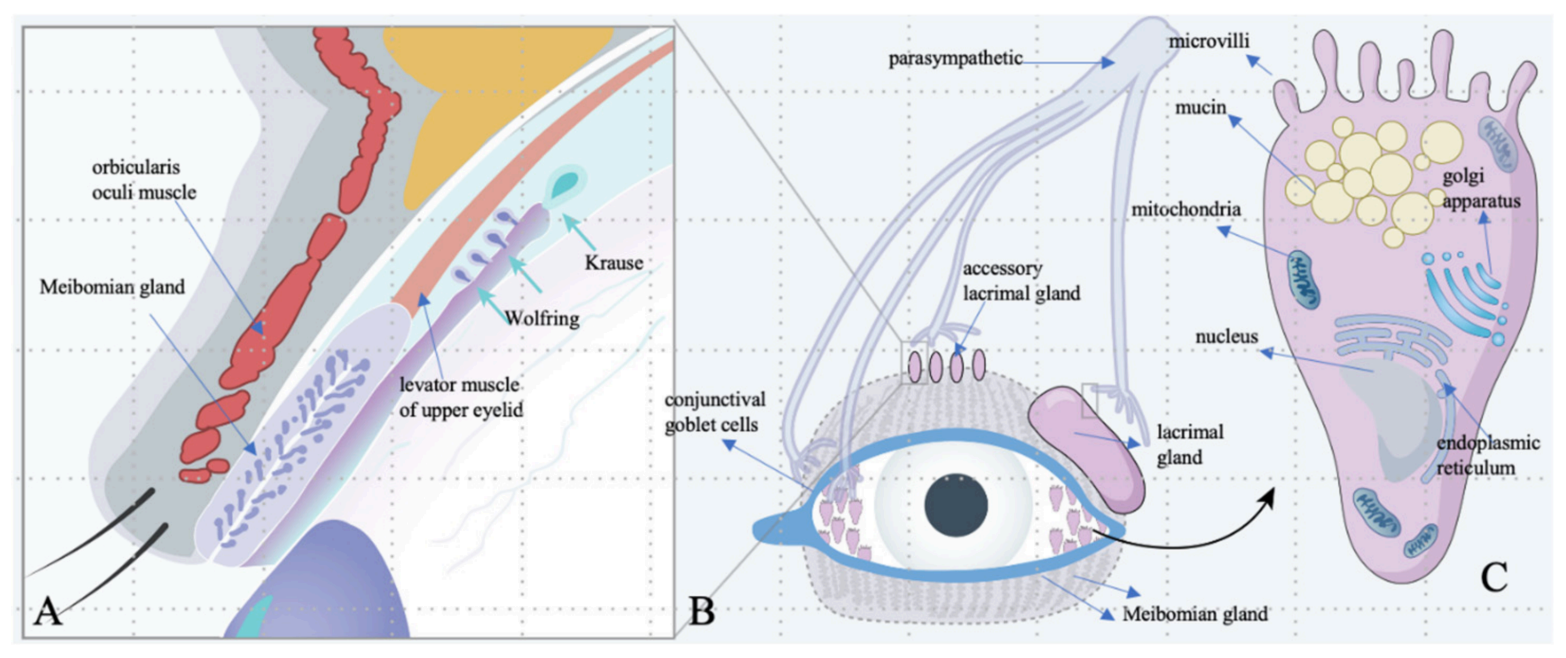
(**A**) Local anatomy of the eyelids. (**B**) The distribution of ocular nerves in the lacrimal gland, accessory lacrimal gland and conjunctival goblet cells. (**C**) Microscopic map of mucin secretion by goblet cells.

Parasympathetic nerves play a critical role in maintaining ocular surface homeostasis via regulating the secretory structures responsible for tear production, including the main and accessory lacrimal glands, as well as the conjunctival goblet cells. Activation of parasympathetic nerves promotes the secretion of aqueous tears and mucins, while inhibition of these nerves leads to reduced secretion of eye tear and manifestation of dry eye symptoms (Figure 3) [5]. Therefore, inhibiting the parasympathetic nerves with anticholinergic agents such as atropine and scopolamine can be a commonly employed method to establish dry eye models [6].

One effective acetylcholine receptor blocking agent that has been used in constructing dry eye animal models is atropine. Atropine exerts anticholinergic effects by competitively inhibiting acetylcholine at muscarinic receptors, thereby suppressing parasympathetic activity and significantly reducing secretion of tear solution from the lacrimal gland and goblet cells [1]. Derivatives of atropine can also be used in constructing the dye eye animal models. Methyl atropine offers advantages such as shortened induction time and suitability for the qualitative evaluation of artificial tear therapies [7]. Another potent anticholinergic agent, scopolamine, can also act on the lacrimal gland and conjunctival goblet cells to inhibit the secretion of tear and mucin [6].

The use of acetylcholine receptor antagonists is a convenient and effective method to induce dry eye models within a short timeframe. However, these models also have limitations. Atropine-induced models may not fully recapitulate the complexity of human dry eye disease, particularly in terms of ocular immune responses [9]. Unlike atropine-induced models, scopolamine-based models are widely utilized to investigate the clinical signs of dry eye and associated ocular surface inflammation. For instance, continuous administration of scopolamine via subcutaneous osmotic pumps in Lewis rats provides a more stable and convenient method for inducing dry eye compared to repeated injections [10]. Additionally, subcutaneous injections of scopolamine in mice have also been used to induce dry eye. This model also provides valuable insights into the mechanisms underlying dry eye via demonstrating the involvement of dendritic cells in disease pathogenesis [8].

### 2.2. Botulinum Toxin (BTX)-Induced Dry Eye Animal Models

Tear secretion is primarily regulated by parasympathetic nerves, hence inhibition of the release of acetylcholine can be a method to lead to dry eye. One neurotoxin that inhibits the release of scetylcholine, botulinum toxin (BTX), can suppress parasympathetic signaling, thereby reducing tear production [11]. Although BTX is used therapeutically to manage epiphora resulting from lacrimal drainage obstruction [15,16], periorbital injection of BTX at high doses can induce dry eye symptoms without significant systemic side effects [17,18,19].

Use of the BTX successfully established dry eye models in CBA/J mice. Injection of BTX significantly suppressed tear secretion [11]. Notably, dry eye symptoms induced by BTX-B (Botulinum Toxin type B) persisted for over one month in mice. This extended duration of the dry eye symptoms in this model can more accurately reflect the chronic nature of dry eye in human, compared to acute models [11]. Owing to the reliability, this BTX-induced model has been proposed as an effective platform for screening potential therapeutic agents for dry eye treatment [20].

### 2.3. Benzalkonium Chloride-Induced Dry Eye Animal Models

Preservatives are commonly employed in eye drops to prevent contamination by microorganisms [21]. However, preservatives like benzalkonium chloride (BAK) can induce significant damage to the ocular surface and cause dry eye. BAK promotes dry eye by disturbing the tear film via reducing the density of goblet cells, inducing squamous metaplasia and apoptosis of conjunctival epithelial cells, as well as disrupting the corneal epithelial barrier [21]. Based on these mechanisms, dry eye models have been successfully established in rats, mice, and rabbits through the topical administration of BAK [12,22,23]. The first BAK-induced rabbit dry eye model was developed in 2008 [22]. Various concentrations of benzalkonium chloride solutions, ranging from 0.01% to 1%, were topically administered to rabbit eyes to optimize the development of a dry eye model. BAK 0.1% is considered the optimal concentration for dry eye modeling [22]. Studies using BAK solutions at concentrations ranging from 0.01% to 1% have shown that 0.1% BAK is optimal for inducing consistent dry eye changes. Notably, the resulting effects on the ocular surface induced by BAK remains irreversible even after cessation of its administration [24]. Although BAK with concentration of 0.1% is considered the optimal concentration for inducing dry eye models, higher concentrations may also be utilized to accelerate the development of the models. A higher concentration (0.2%) of BAK has been applied in New Zealand white rabbits, which significantly shortens the modeling period without causing corneal ulceration or neovascularization [25]. The use of rabbit models is more representative compared to rodents. Rabbits offer anatomical and physiological similarities to humans in terms of eye size, structure, and dry eye pathophysiology, making them particularly suitable for translational research [26]. Owing to the irreversible damage to the cells on the ocular surface, the administration of BAK can also trigger inflammatory responses. Thus, dry eye animal models induced by 0.2% BAK are valuable for investigating the inflammatory pathology associated with dry eye [27]. In addition, such models have been used for assessing the efficacy of various ophthalmic formulations, including artificial tears based on *Bletilla striata* polysaccharide, or *Artemia salina* extracts, and drug delivery systems [28].

### 2.4. N-Acetylcysteine (NAC)-Induced Dry Eye Animal Models

The tear film is essential for maintaining ocular surface lubrication, which consists of three layers: the lipid, aqueous, and mucin layers. The mucin layer serves as the innermost interface that coats the corneal epithelium [29]. The role of ocular mucins in the pathogenesis of dry eye has garnered increasing research interest [30]. N-Acetylcysteine (NAC), a mucin-disrupting agent, acts by reducing disulfide bonds within mucin molecules, thereby decreasing their molecular weight and impairing their structural integrity [31]. This disruption leads to instability of the tear film and loss of homeostasis, ultimately resulting in dry eye. Administering NAC on the ocular surface can serve as a useful dry eye animal model for studying tear film instability resulting from mucin dysfunction.

Topical application of 10% NAC has been shown to induce dry eye in animal models that closely resemble human dry eye, characterized by a reduction in conjunctival goblet cell density and decreased expression of the mucin proteins MUC16 and MUC5AC [13]. However, the relatively high concentration of NAC solution (10%) may cause severe toxicity to the ocular surface [13]. Further investigation is required to evaluate whether lower concentrations of NAC can effectively induce a dry eye phenotype while having improved tolerability and clinical relevance.

### 2.5. Concanavalin a (ConA)-Induced Dry Eye Animal Models

Concanavalin A (ConA), a plant-derived lectin, is a potent stimulator of specific T cell subpopulations [32]. When introduced into the lacrimal gland, ConA triggers a local inflammatory response that suppresses tear secretion, leading to dry eye [14]. Injection of ConA into the lacrimal glands of rabbits has been used to establish a short-term dry eye model, demonstrating that inflammatory mediator accumulation disrupts tear film homeostasis [14]. Furthermore, ConA-induced dry eye models can be employed as a screening model to evaluate the efficacy of drug treatments for dry eyes [14]. However, this model exhibits certain limitations, including the relatively short duration of dry eye symptoms [14]. To extend the duration of symptoms and enhance model stability, B-ultrasound-guided injection of ConA into the lacrimal gland was employed, improving precision and prolonging the inflammatory dry eye phenotype [33].

## 3. Lacrimal Gland Resection-Induced Dry Eye Animal Models

Adequate tear secretion is critical for maintaining stability of the tear film. The important exocrine organ responsible for tear production is the lacrimal gland. Damage or dysfunction of the lacrimal gland can lead to decreased tear secretion, thereby contributing to the development of dry eye disease. The lacrimal glands share similar physiological structures and functions in tear secretion and ocular surface homeostasis in humans and animal [9]. As a result, inducing varying degrees of lacrimal gland tissue damage in animal models may provide valuable insights and methodologies for developing diverse tear-deficient dry eye models that closely replicate the clinical conditions observed in humans. In this section, dry eye models constructed via complete and partial resection of lacrimal glands are introduced. An overview of commonly Lacrimal Gland Resection-Induced dry eye models is provided in Table 2.

### 3.1. Complete Lacrimal Gland Resection

Lacrimal gland resection is one of the methods to develop animal models with severe dry eye [34]. Although the lacrimal glands share similar physiological structures, the histological structure of the glands in various animals exhibit difference from those in humans [34,35]. Precise anatomical localization of the lacrimal glands is essential to ensure their accurate excision.

**Figure 4:**
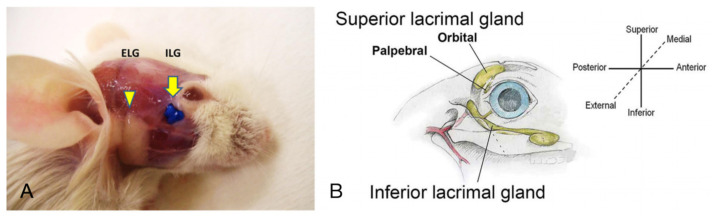
The anatomy of the lacrimal glands. (**A**) ELG and ILG positioning of mouse using dye (arrow) to color the ILG blue. ELG (arrow) exhibits lager size than ILG (reproduced from Ref. [34] with permission from Scientific Reports, Copyright 2018 Scientific Reports). (**B**) Anatomical diagram of upper and lower lacrimal glands of rabbit (reproduced from Ref. [35] with permission from Current Eye Research, Copyright 2019 Current Eye Research).

The exorbital lacrimal gland (ELG) is the main lacrimal gland for rodents. The ELG is located subcutaneously in front of the ear in adult mice, approximately 3 mm from the temporal side of the eye. The intraorbital lacrimal gland (ILG), which is situated beneath the conjunctival bulbar of the outer canthus, is significantly smaller than the ELG (Figure 4) [37]. The complete resection of the lacrimal glands contributes to a dry eye model in LysM mice [34]. Complete resection of both the ILG and ELG resulted in a stronger inflammatory response on the ocular surface and a more prolonged duration of dry eye symptoms [34]. These dry-eye-related symptoms persisted up to 24 weeks after the removal [34].

Unlike rodents, the lacrimal gland in rabbits consists of the superior lacrimal gland (SLG) and ILG. The SLG can be further divided into the orbital superior lacrimal gland (OSLG) and the palpebral superior lacrimal gland (PSLG) (Figure 4) [38]. The SLG is located deep within the medial orbit and cannot be exposed directly through a temporal or lower orbital skin incision (Figure 4) [38]. In contrast, the PSLG is located in the temporal part of the upper eyelid and appears as a small bulge when the upper eyelid is everted (Figure 4) [38]. The ILG is surrounded by abundant blood vessels, and its structure varies considerably among individuals [35]. Consequently, resection of the SLG and ILG in rabbits must be performed sequentially and with caution. Complete lacrimal gland resection has also been used successfully to establish dry eye animal models in rabbits [37]. This approach creates an intensely dehydrated ocular surface microenvironment, providing a robust platform for investigating the pathophysiology of advanced dry eye and evaluating potential therapeutic interventions.

### 3.2. Partial Lacrimal Gland Excision

In addition to total gland removal, partial excision of the lacrimal gland offers an alternative approach for establishing moderate dry eye animal models. For instance, a mouse model of dry eye has been successfully generated by selectively resecting the ELG [36]. Compared to complete lacrimal gland removal, this procedure is less invasive and more easy to operate [34,36]. Inflammation and reduced hydration were exhibited on the ocular surface following the partial ELG. However, the symptoms were generally milder and more transient than those induced by complete resection. These symptoms typically resolve within approximately 14 days with partial removal of ELG [36]. This model is particularly useful for investigating moderate dry eye conditions and well-suited for short-term interventional studies.

## 4. MGD-Related Dry Eye Animal Models

**Figure 5:**
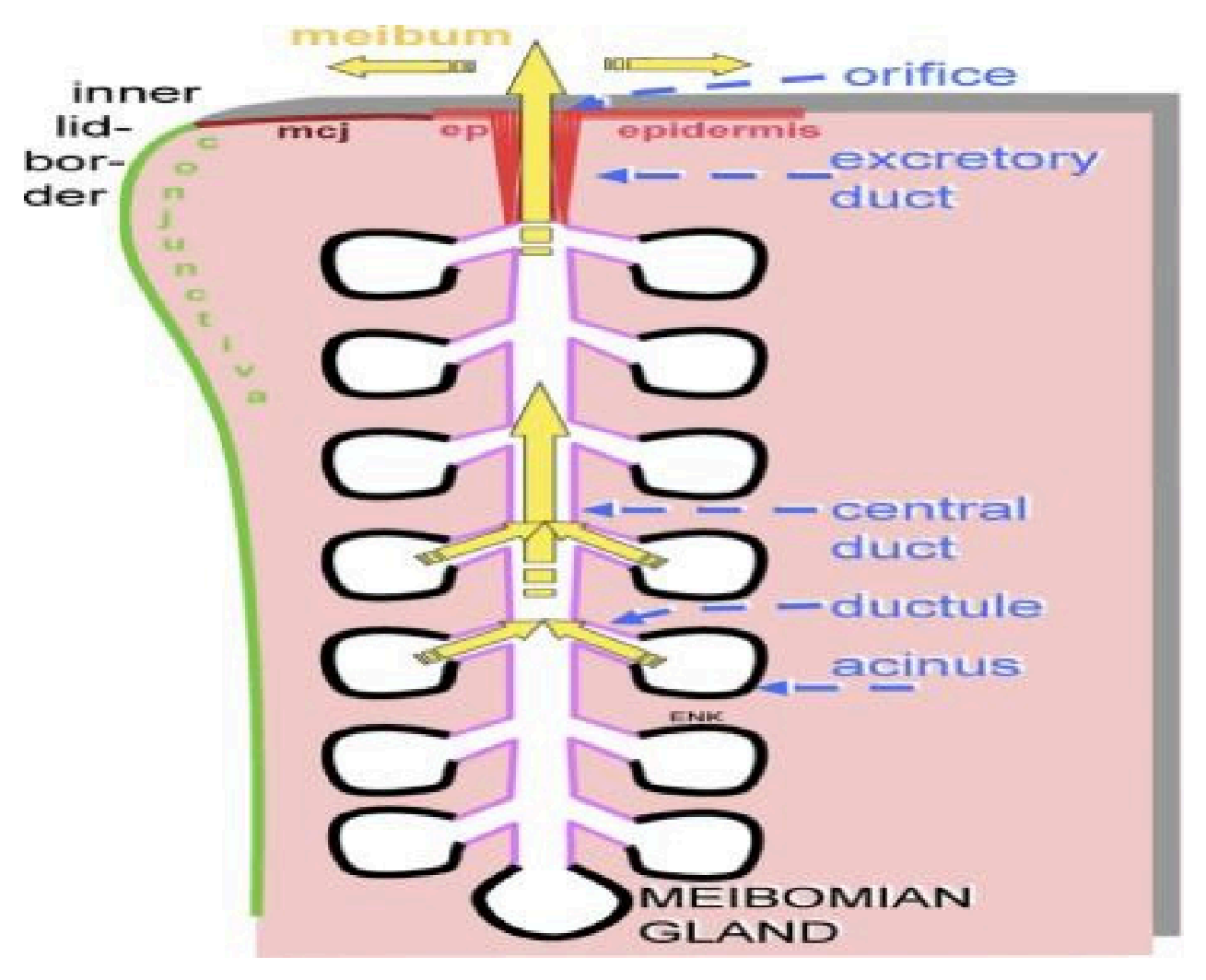
The anatomy of the meibomian gland. The meibomian gland consists of the secretory acine, peripheral duct and central duct (reproduced from Ref. [40] with permission from Springer Science and Business Media, Copyright 2009 Springer Science and Business Media).

**Figure 6:**
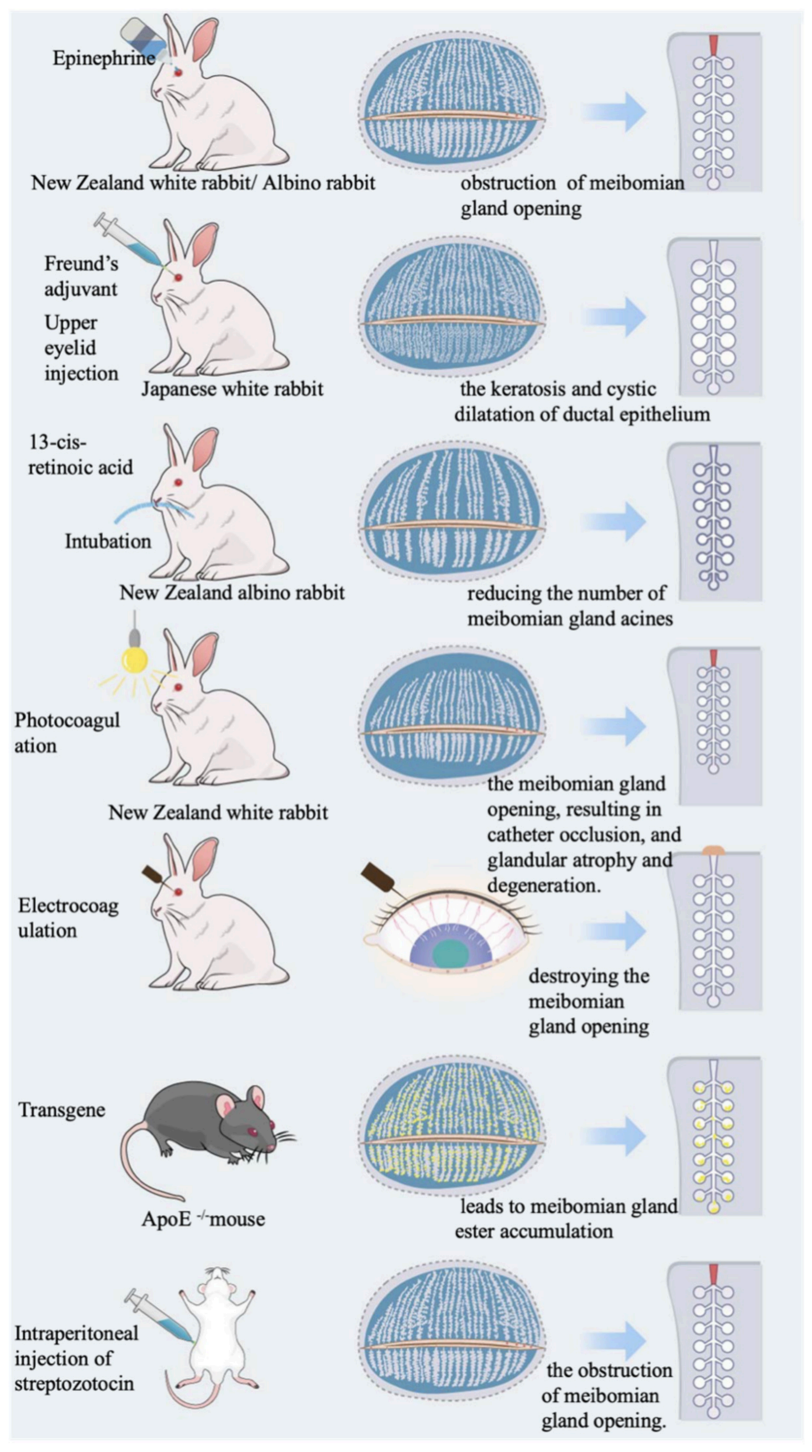
Illustration of meibomian-gland-obstruction-related MGD animal models.

The meibomian glands are situated within the tarsal plates of the upper and lower eyelids and aligned perpendicularly to the eyelid margin (Figure 5) [39]. These glands play a critical role in stabilizing the tear film and preventing dry eye disease. Meibomian gland dysfunction (MGD) has been recognized as the leading cause of evaporative dry eye disease, and has garnered significant attention as a target for developing diverse therapeutic approaches for dry eye management [5]. Animal models of MGD have demonstrated high efficacy in replicating key clinical features of the dry eye condition. The development of diverse MGD modeling approaches supports not only drug screening but also the establishment of pathophysiologically relevant models that more accurately reflect the underlying mechanisms of the disease in humans (see Table 3 and Figure 6).

MGD is a chronic and diffuse disorder of the meibomian glands, commonly characterized by terminal obstruction of the terminal ducts or decreased glandular secretion [40]. This reduction in lipid secretion compromises the integrity of the tear film’s lipid layer, leading to increased tear evaporation and subsequent ocular surface desiccation [40]. MGD has been recognized as a very common cause of dry eye as it is estimated to contribute to over 50% of all dry eye cases [49]. Notably, MGD can be experimentally induced with minimal trauma to surrounding ocular tissues, making it a highly feasible approach for studying dry eye. The availability of diverse MGD animal models offers valuable platforms for investigating the etiology and pathogenesis of MGD-related dry eye. Consequently, inducing dry eye with developing MGD has garnered substantial interest in the field of dry eye research. The following sections will introduce the various methodologies employed to induce MGD in experimental dry eye models.

### 4.1. Drug-Related MGD Animal Models

#### 4.1.1. Epinephrine-Induced MGD Model

Epinephrine can promote hyperkeratinization of the deeper ductal epithelium [42]. The induced excessive keratinization of the ductal epithelium in the meibomian gland can result in orifice obstruction and eventually MGD. The first epinephrine-induced MGD model was established in rabbits. Epinephrine hydrochloride eye drops were topically administered, which led to excessive keratinization and subsequent obstruction of the meibomian gland orifices [41]. A follow-up study in 1989 administered epinephrine hydrochloride eye drops to albino rabbits [42]. Ductal epithelial keratosis and dry eye symptoms persisted for up to six months, confirming that epinephrine-induced keratosis is associated with MGD onset.

#### 4.1.2. Freund’s Adjuvant-Induced MGD Model

Apart from the promoted hyperkeratinization, inflammatory conditions can also contribute to MGD. The inflammatory conditions affect the palpebral conjunctiva and eyelid margin, which can disrupt the structure and function of meibomian glands [50]. Injection of inactivated bacteria into meibomian glands can induce inflammatory response effectively. Complete Freund’s Adjuvant (CFA), a water-in-oil emulsion containing inactivated Mycobacterium tuberculosis, can induce chronic inflammation characterized by immune cell infiltration and cell-mediated immune responses in and around the meibomian glands. CFA-based MGD models are particularly useful for studying the pathophysiology of MGD complicated by meibomian adenitis [43].

#### 4.1.3. Isotretinoin-Induced MGD Model

Inducing cell damage is also employed as a strategy in provoking MGD in animal models. Injection of acidic agents can cause cellular injury within meibomian glands. Isotretinoin (13-cis-retinoic acid), a retinoid widely used in the treatment of severe acne, exerts profound effects on sebaceous glands [51]. However, it inhibits lipid synthesis in sebocytes, suppresses cell proliferation, and induces sebocyte apoptosis [52]. An MGD rabbit model was constructed using systemic isotretinoin administration [44], which led to a reduced number and size of meibomian gland acini and hypertrophy of the epithelium in the central and surrounding ducts. This model provides insight into retinoid-induced MGD and meibomian adenitis, and helps elucidate the relationship between meibomian gland dysfunction and related disorders. Additionally, isotretinoin disrupts meibomian gland cell differentiation via the PPARγ pathway, further contributing to MGD development [52].

### 4.2. Meibomian-Gland-Obstruction-Related MGD Model

Obstruction of the meibomian gland—manifested as orifice blockage, ductal occlusion, and glandular degeneration—represents the most prevalent cause of MGD [40]. Several experimental approaches have been developed to simulate this condition. In 1989, photocoagulation was applied to obstruct the meibomian glands of New Zealand white rabbits [45]. More recently, electrocoagulation has been employed to establish an MGD model in the same species [46]. However, this technique often relies on angiography for guidance, which has inherent limitations. Angiography is unable to detect early inflammatory changes within the meibomian glands, potentially leading to misinterpretation of the initial experimental outcomes and therapeutic effects.

### 4.3. Hyperlipidemia-Related MGD Model

Hyperlipidemia is a well-established risk factor for cardiovascular and peripheral vascular diseases [46,53]. Additionally, it has also been implicated in the pathogenesis of MGD [54]. Patients with MGD often exhibit elevated serum cholesterol levels compared to individuals without MGD, even in the absence of a clinical hyperlipidemia diagnosis [54]. This suggests that dysregulated cholesterol metabolism may contribute to the development of MGD and dry eye syndrome.

Apolipoprotein E (ApoE), a ligand for lipoprotein receptors, is involved in lipid transport and metabolism [55]. In 2019, a hyperlipidemia-related MGD model was established using male ApoE knockout (ApoE ^−^/^−^) mice over a 3-month period. This animal model has provided valuable insights into inflammatory pathways and molecular mechanisms underlying MGD pathophysiology [47].

### 4.4. Endocrine-Imbalance-Related MGD Models

Clinical studies have demonstrated associations between MGD and various systemic factors, including androgen deficiency [56] and diabetes mellitus [1]. Androgen deficiency, in particular, has been recognized as a principal hormonal regulator of meibomian gland function, and its insufficiency is correlated with an increased risk of MGD. Similarly, diabetes mellitus can adversely affect the ocular surface through multiple mechanisms, contributing to the development of dry eye syndrome [57] and recurrent corneal erosion [58]. Individuals with diabetes mellitus are more susceptible to MGD compared to non-diabetic individuals [59]. Patients with type 2 diabetes mellitus (T2DM) are more likely to exhibit tear film instability [48], a reduction in the number of functional meibomian glands, and an upregulation of the inflammatory response (Yu et al., 2016) [48]. These findings have been corroborated in diabetic animals [60]. Four months after intraperitoneal injection of streptozotocin (STZ), rats exhibited orifice blockage and disordered morphology of the meibomian gland [60]. Therefore, the STZ-induced rat models can be considered suitable for investigating the correlation between MGD and abnormal glucose metabolism.

## 5. Environmentally Induced Dry Eye Animal Models

Disruption of tear film homeostasis represents a fundamental pathological mechanism in dry eye disease. As the outermost layer of the ocular surface, the tear film is continuously exposed to external environmental conditions. Variations in environmental parameters—such as temperature, humidity, airflow, light exposure, and pollutant levels—can destabilize the tear film’s delicate balance, thereby promoting the onset and progression of dry eye disease. Consequently, environmental influences are widely recognized as significant risk factors contributing to tear instability and pathogenesis for dry eye [61].

### 5.1. Low-Humidity-Induced Dry Eye Animal Model

Tear film homeostasis is highly sensitive to environmental humidity [5]. In 2005, a wind tunnel for mice was developed to reduce environmental humidity and establish a novel low-humidity-induced dry eye model [62]. This controlled environmental system (CES) enables the creation of a stable and monitorable low-humidity environment. 

### 5.2. Pollution-Induced Dry Eye Animal Model

**Figure 7:**
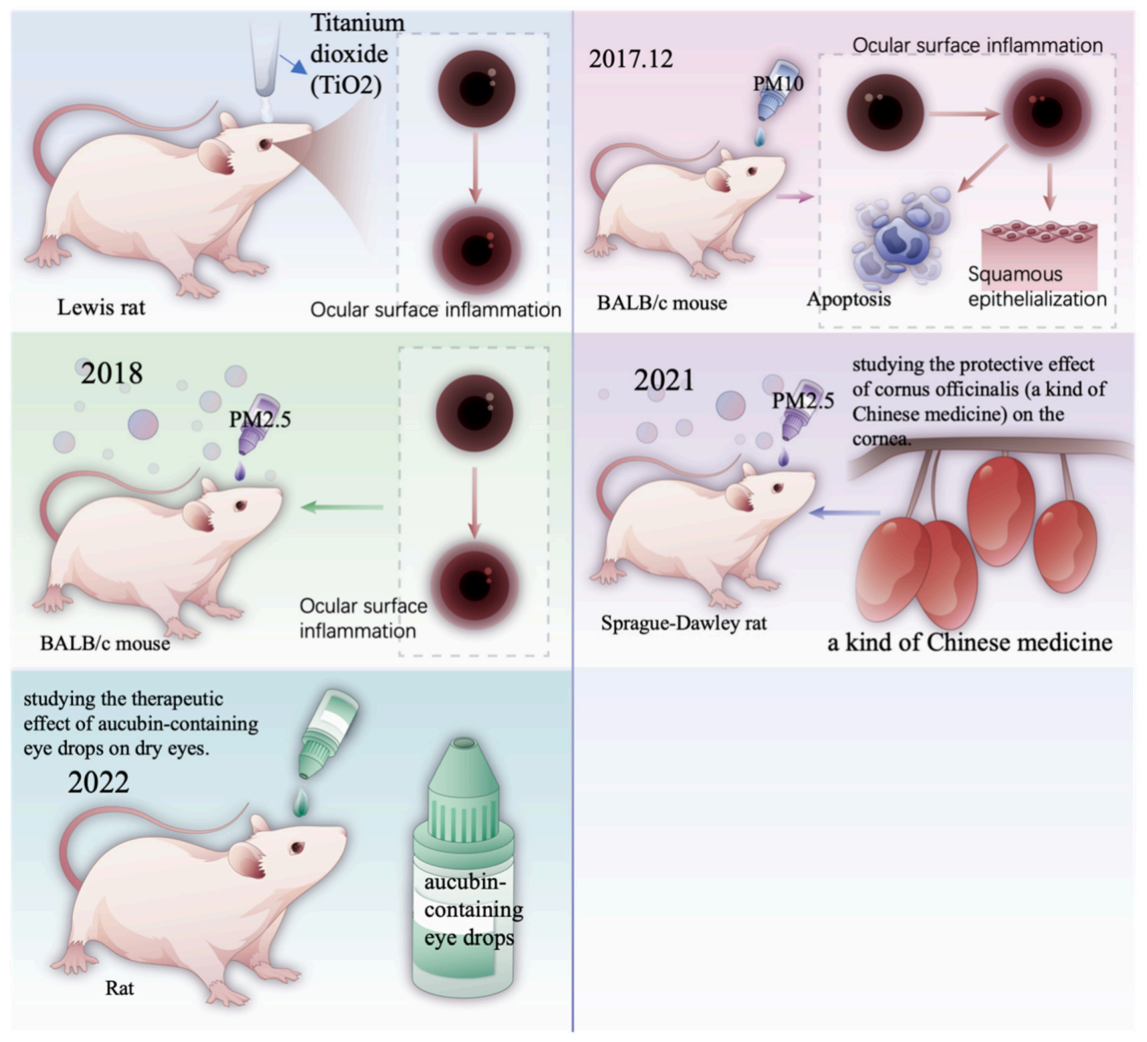
Illustration of animal models with dry eye caused by environmental pollution.

Air pollution is a major global public health concern, which can also contribute to development of dry eye. As a major pollutant, atmospheric particulate matter (PM) comprises liquid and solid particles of varying sizes and chemical compositions. To explore the relationship between PM exposure and dry eye, rat models have been established using topical administration of PM suspensions [63,64]. The PM10-induced rat model exhibited pronounced ocular surface inflammation, apoptosis, and squamous metaplasia—pathological features that closely resemble those observed in human dry eye diseases [63]. Similarly, PM2.5, another key indicator of air pollution severity, has been used to develop dry eye models [65]. A PM2.5-related dry eye model was developed, in which NF-κB expression was upregulated following administration of PM2.5 eye drops, suggesting a potential role of NF-κB activation in the pathogenesis of dry eye. These findings are valuable for evaluating potential therapeutic interventions [65]. Various treatments, including eye drops containing *Cornus officinalis* and aucubin, have been evaluated using the PM2.5-induced dry eye models [66,67]. To better replicate real-world exposure, aerosol-based PM exposure was employed. Compared to local administration via eye drops, aerosol exposure accurately simulates human environmental exposure patterns, thereby offering a more realistic model for studying environmentally induced dry eye (Table 4 and Figure 7) [68].

## 6. Autoimmune-Diseases-Related Dry Eye Animal Models

Sjögren’s syndrome (SS) is a chronic, systemic autoimmune disorder characterized by lymphocytic infiltration and immune-mediated destruction of exocrine glands. In SS, the function of lacrimal and salivary glands are suppressed, which results in symptoms such as dry eye and dry mouth [69]. SS can be classified into primary SS and secondary SS. Primary SS occurs as an isolated condition, while secondary SS often coexists with other autoimmune conditions including rheumatoid arthritis, systemic lupus erythematosus (SLE), systemic sclerosis, autoimmune liver disease, or mixed connective tissue disease [70]. Transgenic mouse models offer a novel approach for investigating autoimmune mechanisms underlying dry eye associated with SS (Table 5).

To recapitulate SS-related pathology, a variety of genetically engineered mouse strains have been employed. The non-obese diabetic (NOD) mouse, which spontaneously develops autoimmune diabetes, is widely used to study autoimmunity-induced dry eye [75]. Lymphocytic infiltration disrupts the extracellular matrix of the lacrimal gland, inducing SS-like symptoms and dry eye in the NOD mouse [71]. The MRL/lpr mouse, generated through multi-generational inbreeding, spontaneously develops a disease resembling human SLE [72]. The lpr mutation affects the Fas antigen, causing lymphoid hyperplasia [73] and making this model suitable for studying dry eye diseases with primary SS [74]. Mice deficient in the DNA-binding inhibitor Id3 (ID3 KO) also display lymphocyte infiltration in the lacrimal and salivary glands. These mice produce diagnostic SS-related autoantibodies, including anti-Ro and anti-La antibodies [76]. As Id3 is an early response gene involved in T cell selection during embryonic development, its deficiency leads to phenotypes that closely resemble primary SS, making Id3-deficient mice a valuable model for studying this form of the disease. Other strains such as NFS/sld [77], IQI/Jic [78], and CD25KO mice have also been used in experimental research. These autoimmune models compromise lacrimal gland function, leading to reduced tear secretion and dry eye symptoms.

## 7. Conclusions

Dry eye is the most common disease on the ocular surface, which can impair vision and cause severe discomfort to patients. Despite its high prevalence and impact on quality of life, the underlying pathophysiological mechanisms remain incompletely understood. Furthermore, current diagnostic and therapeutic strategies are often suboptimal, which contributes to the difficulty of dry eye management. Animal models with dry eye have attracted attention for investigating the pathophysiology, diagnosis and treatment of the disease. This review has summarized a range of dry eye animal models, including drug-induced, lacrimal gland resection, MGD, autoimmune, and environmental-induced models. Table 6 provides a comparative framework for selecting appropriate models based on specific experimental requirements, such as induction duration and methodological considerations. However, dry eye can arise from diverse ocular pathologies, and animal models generated under distinct etiologies are typically restricted to particular subtypes. Anticholinergic treatment produces a model characterized by tear film instability and reduced aqueous layer, whereas NAC administration induces mucin-layer disruption. BAK-induced models are more closely associated with corneal epithelial damage and inflammation, while lacrimal gland excision yields a severe aqueous-deficient model. MGD-based models represent evaporative dry eye, and autoimmune strains serve as models for Sjögren’s syndrome-associated dry eye.

While this review categorizes five distinct model types according to induction methods, species used, advantages, and limitations, it does not provide an in-depth comparative analysis of the underlying pathophysiological mechanisms of each model. Although various in vitro models have been explored for dry eye research, animal models remain an excellent platform for elucidating disease mechanisms, as they provide the most reliable mimicry of the in vivo microenvironment. Future research should focus on elucidating these mechanistic differences to better inform model selection and improve the translational relevance of preclinical findings in dry eye disease. An overview of dry eye models is provided in Table 6.

## 8. Search Strategies

Comprehensive literature searches were conducted on three electronic databases including PubMed, Embase (Ovid) and Web of Science Core Collection as well as hand-searching of relevant journals and reference lists of included studies. The key words searched for in the literature included “dry eye” and “animal models”. Databases were searched from inception (1 November 1978) to the date of the search. The inclusion criteria were predetermined and guided by the selection of references to ensure that only studies meeting specific eligibility criteria were included. A total of 38 articles were related to the establishment of animal models and were included in this review. Different kinds of animal models were summarized in tables which recorded the advantages or disadvantages of the animal model in detail. Only the literature discussing the establishment of animal models or study of dry-eye-related disease with novel animal models were included in this review. In addition, the selection process also involved the assessment of the methodological quality of the identified references to ensure that only reliable and valid evidence was included in the review. This selection process was designed to provide a comprehensive overview of the current landscape of dry eye animal models.

## Figures and Tables

**Table 1:** Drug-related dry eye animal models.

Drug	Administering Method	Mechanisms	Animal	Advantage	Disadvantage	Ref.
Acetylcholine receptor blocking agent		Inhibits parasympathetic excitation of lacrimal gland and conjunctiva				
Atropine	Eye drops		New Zealand white rabbit	1. Shorter modeling time 2. Ability to evaluate artificial tears	Inaccurate and incomplete replication of human dry eye pathology	[9]
Scopolamine	Subcutaneous osmotic pump		Lewis’s rat	Similar pathological state to human dry eye	Needs to further improve the diagnostic index	[10]
Scopolamine	Subcutaneous injection		C57BL/6 mouse	Demonstration of dendritic cell-mediated immunoinflammatory response in inducing dry eye		[8]
BTX	Lacrimal injection	Blocks acetylcholine in neuromuscular and cholinergic nerve	CBA/J mouse	Prolongs duration of dry eye conditions more than 1 month		[11]
Benzalchloramine	Eye drops	Destructs the goblet cells from ocular surface	New Zealand white rabbit	1. Stable pathological model of dry eye 2. Similar pathophysiological mechanisms to human dry eye	Insufficient study of the recovery process after withdrawal of benzalkonium chloride	[12]
NAC	Eye drops	Destroys the molecular structure of mucin	Sprague-Dawley rat	Similar features to human dry eye		[13]
ConA	Lacrimal injection	1. Stimulates the production of T cells 2. Aggravates the ocular surface inflammation	New Zealand white rabbit	Efficient construction of the dry eye animal model within a reduced timeframe		[14]

BTX = botulinum toxin; NAC = N-acetylcysteine; ConA = Concanavalin A.

**Table 2:** Dry eye animal models associated with lacrimal gland resection.

Operation Mode	Mechanism	Animal Type	Anesthesia Mode	Advantage	Disadvantage	Ref.
Complete lacrimal gland resection	Completes lacrimal gland resection can lead to insufficient tear production	LysM mouse	Intraperitoneal injection of ketamine and cyrazine	Prolongs duration of dry eye conditions	Difficulty in surgery operation	[34]
		New Zealand white rabbit	Subcutaneous injection of lidocaine	Construction of an extremely dry environment on ocular surface	Difficulty in surgery operation	[35]
Partial lacrimal gland excision	Partial lacrimal gland excision can cause insufficient tear production	C57BL/6 mouse	Intraperitoneal injection of ketamine and cyrazine	Simplified surgery operation	Transient duration	[36]

**Table 3:** MGD-related dry eye animal models.

Animal Modeling Approach	Mechanisms	Administering Method	Animal	Advantage	Disadvantage	Ref.
Drug-related MGD animal model						
Epinephrine	Promotes excessive keratinization of the duct epithelium of the meibomian gland	Eye drops	New Zealand white rabbit	The first epinephrin-related MGD model in rabbits		[41]
	Promotes excessive keratinization of the duct epithelium of the meibomian gland	Eye drops	New Zealand white rabbit	Determination of the earliest changes in adrenergine-induced MGD	Unclear effect of keratosis on meibomian gland function	[42]
Freund’s adjuvant	Promotes the inflammatory response of meibomian gland	Upper eyelid injection	Japanese white rabbit	Establishment of a novel MGD animal model simulating human meibomian gland inflammation	Reflects short-term changes in inflammatory response associated with dry eye	[43]
13-cis-retinoic acid	1. Reduces the number of meibomian gland acines 2. Promotes the thickening of the central duct	Intubation	New Zealand albino rabbit	Understands the pathogenesis of palpebral conjunctivitis		[44]
Meibomian-gland-obstruction-related MGD animal model						
Photocoagulation	Destroys the meibomian gland orifices		New Zealand white rabbit	Support of MGD as the cause to dry eye		[45]
Electrocoagulation	Destroys the meibomian gland orifices		New Zealand white rabbit	Exploration of mechanism of MGD damaging ocular surface	Limitation in observing early inflammatory changes in meibomian gland via angiograph	[46]
Hyperlipidemia-related MGD animal model	Elevated blood lipids can cause the obstruction of meibomian gland orifices	Transgene	ApoE ^−^/^−^ mouse	Application of ApoE ^−^/^−^ mice for relationship between hyperlipidemia and MGD		[47]
Endocrine-imbalance-related MGD animal model	Elevated blood sugar can cause the obstruction of meibomian gland orifices	Intraperitoneal injection of streptozotocin	Sprague-Dawley rat	Ability to study the relationship between the MGD and abnormal glucose metabolism		[48]

**Table 4:** Animal models with dry eye caused by environmental pollution.

	Animal	Method	Advantage	Ref.
Titanium dioxide (TiO_2_)	Lewis’s rat	Applying conjunctival sac locally	Novel application of PM-inducing dry eye rat model	[64]
PM10	BALB/c mouse	Using eye drops	Contribution to significant ocular surface inflammation, apoptosis, and squamous epitheliosis	[63]
PM2.5	BALB/c mouse	Using eye drops	Verification of the relationship between the NF-κB activation and dry eye progression	[65]
PM2.5	Sprague-Dawley rat	Using eye drops	1. Evaluation of the protective effect from cornus officinalis	[67]
PM	Rat	Using eye drops	2. Ability to measure therapeutic effect of aucubin-containing eye drops on dry eyes	[66]
PM	Sprague-Dawley rat	Using aerosol	Similar polluted environment to humans	[68]

**Table 5:** Dry-eye-related transgenic mouse.

Mouse	Features	Ref.
NOD mouse	A spontaneous model of both type 1 diabetes mellitus (T1DM) and SS	[66]
MRL/lpr mouse	1. Derived from multi-generation hybridization of closely related mice 2. Similar to human SLE 3. Used in the study of primary SS	[70]
Id3-deficient mouse	1. Detects with anti-ro and anti-La antibodies (specific antibodies for the diagnosis of SS) in its serum 2. Used in the study of primary SS	[71]
NFS/sld mouse	Uses a thymectomized mouse with SS appearing three days after birth	[72]
IQI/Jic mouse	Shows focal lymphocyte infiltration with parenchymal destruction	[73]
CD25KO mouse	Observes the T cell gene expression in the cornea and conjunctival epithelium	[74]

Sjogren’s syndrome = SS; type 1 diabetes mellitus = T1DM.

**Table 6:** Summary of different dry eye animal models.

Type	Administration Mode	Animal	Molding Period	Ocular Surface Steady State Evaluation Index	Lacrimal Gland-Related Research	Ref.
Drug type						
Acetylcholine receptor blocking agent						
Atropine	Using eye drops	New Zealand white rabbit	5 days	1. Ferning test 2. The Schirmer test 3. Fluorescein staining		[9]
Scopolamine	Subcutaneous osmotic pump	Lewis’s rat	28 days	1. Fluorescein staining 2. MHC II expression and mucin Muc5AC production		[10]
Scopolamine	Subcutaneous injection	C57BL/6 mouse	2 days	Fluorescein staining		[8]
BTX	Lacrimal injection	CBA/J mouse	28 days	1. Phenol red thread test 2. Fluorescein staining	Hematoxylin and Eosin staining of the lacrimal gland	[11]
Benzalkonium chloride	Using eye drops	New Zealand white rabbit	14 days	1. Fluorescein and Rose Bengal Staining 2. Schirmer test 3. MUC5AC staining in cornea		[12]
NAC	Using eye drops	Sprague-Dawley rat	5 days	1. Fluorescein and Rose Bengal Staining 2. Phenol red thread test 3. Concentration of MUC5AC in tears 4. Goblet cells from conjunctival epithelium		[13]
ConA	Lacrimal injection	New Zealand white rabbit	1 day	1. Tear breakup time assessment 2. Schirmer test	1. Hematoxylin and Eosin staining of the lacrimal gland 2. Lacrimal gland cytokine and MMP-9 levels	[14]
Operation mode						
Complete lacrimal gland resection		LysM mouse	14 days	Fluorescein staining		[34]
Partial lacrimal gland excision		C57BL/6 mouse	14 days	1. Fluorescein staining 2. Phenol red thread test 3. Real-Time Polymerase Chain Reaction	Hematoxylin and Eosin staining of the lacrimal gland	[36]
Drug-related MGD animal model						
Epinephrine	Using eye drops	New Zealand white rabbit	6 months	Light micrograph of normal meibomian gland		[42]
Freund’s adjuvant	Upper eyelid injection	Japanese white rabbit	29 days	Hematoxylin and Eosin staining of the meibomian gland		[43]
13-cis-retinoic acid	Intubation	New Zealand albino rabbit	3 months	Light micrograph of normal meibomian gland		[44]
Meibomian-gland-obstruction-related MGD animal model						
Photocoagulation	Photocoagulation	New Zealand white rabbi	12 weeks	1. Rose Bengal staining of the cornea 2. Schirmer test		[45]
Electrocoagulation	Electrocoagulation	New Zealand white rabbit	14 weeks	1. Phenol red thread test 2. Fluorescein staining 3. Hematoxylin-eosin (HE) staining of the sections of meibomian gland		[46]
Hyperlipidemia-related MGD animal model	Transgene	ApoE ^−^/^−^ mouse	3 months	1. Fluorescein staining 2. Oil red o staining 3. Immunohistochemical staining 4. Tunnel assay		[47]
Endocrine-imbalance-related MGD animal model	Intraperitoneal injection of streptozotocin	Sprague-Dawley rat	4 months	1. Hematoxylin-eosin (HE) staining of the sections of meibomian gland 2. Oil red o staining 4. Immunofluorescent staining 4. Immunohistochemical staining 5. Tunnel assay		[48]
Environmental modeling method						
Titanium dioxide (TiO_2_)	Applying conjunctival sac locally	Lewis’s rat	24 h	1. Phenol red thread test 2. Concentration of MUC5AC in tears 3. Inflammatory cell infiltration		[64]
PM10	Using eye drops	BALB/c mouse	14 days	1. Fluorescein staining 2. Phenol red thread test 3. Rose Bengal staining 4. Immunofluorescent staining		[63]
PM2.5	Using eye drops	BALB/c mouse	14 days	1. Fluorescein staining 2. Schirmer tear test 3. Phenol red thread test 4. Tunnel assay	Hematoxylin and Eosin staining of the lacrimal gland	[65]
PM2.5	Using eye drops	Sprague-Dawley rat	14 days	1. Phenol red thread test 2. Hematoxylin and eosin staining 3. Periodic acid-schiff staining 4. Tunnel assay	Hematoxylin and Eosin staining of the lacrimal gland	[67]
PM	Using eye drops	Sprague-Dawley rat	5 days	Phenol red thread test	1. Levels of ROS in the lacrimal gland 2. Tunnel staining	[66]
PM	Using aerosol	Sprague-Dawley rat	14 days	1. Phenol red cotton thread 2. Fluorescein staining		[68]

## Data Availability

No new data were created or analyzed in this study. Data sharing is not applicable to this article.
